# Factors associated with self-management in older adults with multiple chronic conditions: a qualitative study

**DOI:** 10.3389/fpubh.2024.1412832

**Published:** 2024-09-13

**Authors:** Hajar Sadeghi, Farahnaz Mohammadi Shahbolaghi, Mohammadali Hosseini, Masoud Fallahi-Khoshknab, Gholamreza Ghaedamini Harouni

**Affiliations:** ^1^Nursing Department, Student Research Committee, University of Social Welfare and Rehabilitation Sciences, Tehran, Iran; ^2^Nursing Department, Iranian Research Center on Aging, University of Social Welfare and Rehabilitation Sciences, Tehran, Iran; ^3^Social Welfare Management Research Center, Social Health Research Institute, University of Social Welfare and Rehabilitation Sciences, Tehran, Iran

**Keywords:** older adults, chronic conditions, self-management, qualitative content analysis, chronic disease

## Abstract

**Background and purpose:**

Recognizing the importance of self-management in older adults with multiple chronic conditions (MCCs) is crucial for their quality of life. This qualitative study explored the factors linked to self-management among older adults with MCCs.

**Materials and methods:**

The present study was conducted in three stages: an integrated review, qualitative interviews, and Delphi. The search used electronic databases including Web of Science, PubMed, Scopus, Magiran, SID, and Iranmedex. The results of 33 studies that met the inclusion criteria were analyzed using conventional content analysis. A data matrix was formed; and purposeful sampling was conducted among older adults with MCCs, family caregivers, and specialists. The data were collected through semi-structured interviews. Data analysis of 29 interviews was conducted simultaneously with data collection using oriented qualitative content analysis and the Elo and Kyngäs approach. Three rounds of Delphi were conducted via email correspondence with a group of 30 experts to develop and validate the proposed variables.

**Results:**

The factors that influence self-management can be categorized into various categories. Biological factors, cognitive factors, co-morbidities, socio-economic factors, health-related behaviors, mental health, interactions with healthcare teams, Family relationships, medical facility resources, employee empowerment, health policy development, and cultural influences.

**Conclusion:**

Self-management in older Iranian adults with MCCs is a complex and multidimensional phenomenon. By identifying the relevant factors, it is possible to design operational plans that promote self-management among the older adult population and are tailored to fit the specific needs of Iranian society.

## 1 Introduction

Aging is an inevitable process, and older adulthood population is a global issue that presents a significant challenge to humanity (1). According to United Nations estimates, the global older adult population is expected to double by 2050, that is 1.2 billion people, and Iran ranks third in the world in terms of the speed of population aging, following the UAE and Bahrain (2, 3). Population aging is a reality in Iran, with older adults representing approximately 10% of the population (4). By 2050, the share of this age group in the country’s population is projected to exceed 20%, totaling over 20 million people (5).

As individuals age, the burden of disease and the number of people suffering from multiple chronic conditions (MCCs) also increases. Individuals over the age of 60 have at least one chronic disease (6). Chronic conditions are medical conditions that require ongoing treatment for a year or longer, often resulting in limitations in daily activities. Individuals with MCCs have two or more chronic diseases simultaneously (7). The prevalence of having two chronic diseases at the same time is 58.2% in the 45–64 age group and 78.9% in those over 65 years old (8). A study by Ahmadi et al. in the Golestan province of Iran found that the prevalence of MCCs was 30.4% in individuals aged>60 years. Risk factors for MCCs include being over 60 years old, female, unemployed, a smoker, alcohol or drug user, having low education, physical inactivity, poor socioeconomic status, and a high body mass index (9). Common patterns of MCCs include cardiovascular and metabolic diseases, mental health issues, and musculoskeletal disorders (10).

MCCs increase the need to use healthcare services and facilities, and proper management is a major public health challenge that helps control disease complications and treatment costs (11). Additionally, there is an increase in disability, a decrease in activity levels, an increase in the burden of disease, and a decrease in the feeling of well-being, all of which weaken the quality of life (12) and increase the risk of death (13). To avoid a deterioration in functional capacity, participation in physical activities can positively impact the quality of life and functional capacity of older adults and help maintain independence in basic daily activities. Additionally, living alone can affect basic activities and functional capacity (14). In the context of older adults’ health, it is essential to understand the determinants that influence a high degree of satisfaction with their health status. Key factors include maintaining good functional abilities, the absence of physical diseases and psychological problems, and maintaining adequate levels of physical activity (15). In addition to management and clinical care, it is important to pay attention to the self-management needs of older adults with MCCs to reduce disease symptoms and maintain their quality of life. Currently, healthcare providers for this group of individuals primarily focus on life-threatening conditions and complications to reduce mortality. However, for the multifaceted and comprehensive management of MCCs in older adults, coordination among care plans, disease management, and self-management is crucial. This coordination depends on the patient’s ability and self-care capacity.

In a review of guidelines related to the clinical management of individuals with MCCs, regular evaluation of treatment conditions, self-management, monitoring, and follow-up were considered (16). Self-management is defined as the ability of individuals to manage symptoms, treatment, and physical and psychological consequences of the disease and adapt their lifestyle to accommodate chronic diseases (17). The five main self-management skills include problem-solving, decision-making, correct use of information sources, forming a relationship between the patient and healthcare providers, and Taking Action (18). In recent years, special attention has been paid to self-management strategies, particularly in the management of chronic conditions (19). Self-management is a practical nursing (20). Self-management strategies in individuals with chronic diseases will empower the patient to play an active role in managing the disease, increasing awareness, improving understanding of the disease and adherence to the treatment regimen, enhancing the health status of patients, and reducing the costs of treatment and care (21).

Self-management is one of the most crucial factors in the field of geriatrics (22) and is also a key strategy to reduce the costs of treating and caring for patients, particularly those with chronic illnesses. Health policymakers place great importance on enhancing and refining their self-management skills. When older adults’ self-management abilities are compromised, they may feel powerless and lack decision-making authority. They may feel compelled to follow other decisions and choices, which goes against their natural inclinations. This can compromise autonomy and independence. Furthermore, older adults with limited self-management skills are more susceptible and require additional support to adapt to their circumstances (23). To break this cycle, it is essential to identify the factors that influence and predict self-management in older adults to enhance their self-management abilities (22). Therefore, research and programs aimed at improving self-management among older adults based on their unique circumstances, capabilities, and needs are top priorities in healthcare. Reducing the costs of treating and caring for patients, particularly those with chronic illnesses, is also a key strategy. Health policymakers place great importance on enhancing and refining their self-management skills. Although numerous studies have been conducted on factors associated with self-management, few studies have specifically focused on older adults (17, 23–26).

Previous studies have investigated self-management in older adults with chronic diseases such as rheumatoid arthritis, asthma, and diabetes (27, 28). Since the incidence rate of MCCs is high and its consequences affect self-management differently in older adults than in other age groups (17, 29, 30), it is necessary to analyze the factors related to self-management in this group of patients separately. Given the challenges posed by chronic diseases in older adults and the crucial role that self-management plays in mitigating the severe complications of MCCs, it is essential to explore the factors that influence self-management in this population. As the existing knowledge on this topic is limited, further research is needed for a more comprehensive understanding. Therefore, this study aimed to elucidate factors associated with self-management among older Iranian adults with MCCs.

## 2 Materials and methods

### 2.1 Study design, sample size, and data collection

This study is part of a sequential-exploratory mixed-method study aimed at designing and evaluating a predictive model of self-management in Iranian older adults with MCCs. The research methodology consisted of three parts. First, an integrated literature review is conducted. The second part involved a field study based on interviews with older adults with MCCs, their caregivers, and healthcare specialists (physicians, nurses, and other healthcare team members). The third part involved validating the proposed variables; through three rounds of Delphi, conducted via email correspondence with 30 experts. The integrated literature review follows Whittemore’s approach. The review was conducted by identifying the problem, collecting data, reviewing the literature, evaluating and extracting data, analyzing data, extracting concepts, and interpreting and presenting results (31, 32).

A review of English and Persian research articles was conducted using quantitative and qualitative methods from 1980 to 2023. The inclusion criteria were full-text Persian and English studies with various quantitative and qualitative designs related to self-management and its influencing factors in older adults with MCCs.

A detailed search was performed using English keywords from MESH, such as Multiple chronic conditions, aging, older adults, Self-Management, multi-morbidity, Comorbidity, Concurrent multiple medical conditions, Predictors, Older Adult, Old age, Risk Factor, as well as the keywords Factors, Risk Factors, Predictors. Electronic databases, such as Web of Sciences, PubMed, Scopus, and equivalent Persian databases, including Magiran, SID, and Iranmedex, were used for the search. Studies involving non-older adults or older adults without MCCs were excluded from analysis. Initially, two researchers independently reviewed the abstracts of the articles, followed by a thorough evaluation of the related articles. A total of 33 articles were analyzed after a complete review of their texts. The screening and selection process adhered to PRISMA guidelines (Chart No. 1). To ensure article quality, a five-item checklist by Hong was used (107) and articles were independently graded as excellent (25 articles), with some limitations (eight articles), or with significant limitations. Data analysis was conducted using conventional content analysis.

In the second phase, individual interviews were conducted with the participants based on the matrix obtained from the integrated literature review (Table 1). Participants were selected through purposive sampling; and included healthcare professionals (e.g., physicians, nurses, clinical psychologists, physiotherapists, social workers, pharmacists, and gerontologists), all of whom had experience working with older adults with MCCs. Older adults with a history of MCCs, as confirmed by a physician for at least the last 6 months, living in Tehran City either alone or with family (community-dwelling), and without cognitive impairments (scoring seven or higher on Hodgkinson’s short cognitive test) (33), were required to provide informed consent to participate in the study.

**Table 1:** Characteristics of participants in interviews.

Number of participants in each group	Interviewed group	Age range (year)	Gender		Job number	Participant’s numbers
			Female	Male		
1–13	Specialists	36–50	4	9	Clinical psychologist (1), gerontology specialist, physician (2), gerontology specialist - faculty (3), pharmacist (1), nurse (4), social worker (1), physiotherapist (1)	1–13
14–19	Caregivers	28–51	3	3	Employee (3), faculty member (1), freelance (1), student (1)	14–19
20–26	Older adults with MCCs	65–81	4	3	Retired (3), manual worker (2), housewife (2)	20–26

**Figure 1:**
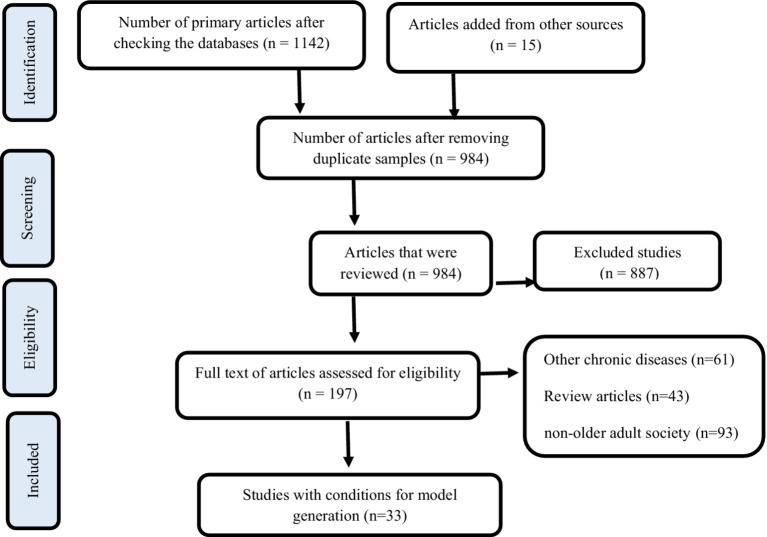
PRISMA flow diagram.

They also needed to be referred to healthcare centers affiliated with Iran, Tehran, and the Shahid Beheshti Universities of Medical Sciences. Additionally, family caregivers of older adults, who were responsible for caring for them at home for at least 6 months and lived with them or separately, also had to provide informed consent to participate in the study. Data were collected from January to June 2023 at clinics, hospitals, and universities in Tehran. After receiving approval and obtaining an ethics code, researchers visited the centers and conducted semi-structured individual interviews with participants after explaining the research and obtaining consent. A total of 29 interviews, were conducted and recorded in a private setting by the first author (Figure 1).

Experts asked open and general questions such as: “Based on your experience of caring for and interacting with older adults with MCCs, what problems have the diseases caused for the older adults?” “, “How can they maintain their health with so many diseases?,” “How do they control problems and complications related to diseases together?,” “What factors help in doing these tasks?”. Caregivers also answered open and general questions, including; “What diseases does your mother, father, or older adults suffer from?”; “Has it created problems for them?,” “How have they maintained their health with so many diseases? “, “How do they control disease-related problems and complications related to diseases?’, “What factors help with doing these things? “).

Interviews with the older adults with MCCs included general questions like (“What diseases are you currently suffering from?,” “What problems have the diseases caused you?,” “How do you deal with the problems and complications related to the diseases?,” “Do you control together?” and “What factors help in doing these things?”). Exploratory questions were also asked to encourage participants to recount their experiences and obtain more information. The interviews were continued based on the initial answers and interview guide regarding self-management. The duration of the interviews was adjusted based on the participants’ opinions and tolerance levels (average 46 min). Interviews continued until data saturation was reached; meaning that no new findings were added.

In the third part, the Delphi technique was used to reach a consensus on the predictors of self-management in older adults with MCCs. A research team comprising experts in chronic diseases, nursing, and geriatrics reviewed the method. The questionnaire, which was administered in three rounds, was emailed to 35 experts between August and November 2023. Thirty experts assessed the importance of each indicator using a five-point Likert scale (completely agree = 5, agree = 4, disagree = 3, completely disagree = 2, and no opinion = 1).

### 2.2 Data analysis

During the integrated review of the articles, traditional content analysis was performed using the Elo and Kyngäs methods. Initially, the results of the articles were examined to extract, code, and classify the factors that influence self-management. Subsequently, the interviews were analyzed using a guided content analysis approach after recording and implementation. The resulting codes were then combined and developed in a conceptual matrix derived from the literature review. Any new codes identified led to the addition of new subcategories to the data analysis matrix. The method outlined by Elo and Kyngäs (108) was employed for this stage with the following steps.

1. Preparation stage: Reading the general text of the data repeatedly to gain a comprehensive understanding and select the analysis unit. Decision-making for data analysis was carried out using either explicit or latent content analysis. 2-Organization stage: open coding and creation of sub-categories, grouping of sub-categories into more abstract categories, and compiling a general description using sub-categories and categories. 3-Reporting phase: Reporting the analysis process and study results through the conceptual model, highlighting inter-conceptual connections (34).

After compiling the data analysis matrix obtained from the post-implementation integrative review of literature, the interviews were read multiple times. By immersing myself in the data. I gained a general understanding of the interview texts. The coding process followed, with white codes and then classified based on similarities and differences within the matrix classes. The Delphi section analyzes prioritized factors related to self-management using descriptive statistics.

### 2.3 Trustworthiness and ethical considerations

Guba and Lincoln’s criteria, including acceptability, reliability, transferability, and verifiability, were used to ensure the scientific accuracy of this study (35, 36). The researcher aimed to establish a proper relationship with the participants and enhance credibility through long-term involvement and complete immersion in the data (37). The findings were verified by examining their compatibility with their experiences and by controlling the data results with them during fieldwork.

In this study, the interviews and extracted codes were presented to several colleagues for analysis to confirm the researcher’s perceptions. Through the analysis process, our colleagues discussed the findings. Researchers have 10–30 years of experience in theoretical and clinical training on chronic diseases and in working with older adult patients with MCCs. In this study, the triangulation of the researcher involved more than one researcher participating in collecting, analyzing, or interpreting the data, which helped increase the credibility of the qualitative aspect of the study.

Triangulation of the data sources included participants with different levels of experience (older adults, caregivers, and experts) selected for data collection. Reliability: The key factor in achieving reliability is the validation of the researcher’s findings.

To ensure reliability, all interviews were recorded and transcribed verbatim. The steps taken were meticulously documented, and the data were stored securely. Additionally, to meet these criteria, the methods were presented to experts for review, and supervisors provided feedback. Finally, when writing the research report, coding was used; and participants’ statements were quoted. Verifiability is a gradual, continuous process. To validate the researcher’s efforts, it was essential to accurately document and report the research steps and decisions made during the process, enabling others to replicate the study, if necessary. To ensure the transferability of the findings, the researcher made a concerted effort to thoroughly describe the research field by outlining the participants, sampling method, and the time and location of data collection. This approach allows readers to have confidence in the potential transferability of the data.

Permission to conduct the research was obtained from the ethics committee of the University of Social Welfare and Rehabilitation Sciences under the code of ethics (IR.USWR.Rec.1399.256). Informed consent was obtained by explaining the purpose of the research to the participants, assuring them that their information (names, interview tapes, and writings) would be kept confidential and that they were free to withdraw at any stage of the research. Recorded conversations were deleted after data transfer and analysis.

## 3 Results

In the first part of the study, codes extracted from the studies in the categories included biological factors, cognitive factors, coexisting diseases, economic and social factors, health-oriented behaviors, mental health, interaction with the care and treatment team, family relationships, medical center facilities, and staff empowerment. Health policymaking and cultural factors are also considered. The codes obtained from the interviews with the participants were categorized based on similarities and differences in the matrix categories from the literature review. Codes that did not fit into any sub-categories formed new sub-categories and primary sub-categories were identified using an inductive approach. Sub-categories obtained from individual interviews included; fatigue, sensitivity and perceived severity of disease complications, happiness, personality type, and caregiver burden (Table 2). In the Delphi stage, individual knowledge of health literacy, treatment cost with care cost, and complex treatment patterns with disease severity were integrated. Variables related to the use of self-management programs were eliminated.

**Table 2:** Categories and subcategories derived from the integrated literature review and stakeholder interviews.

Main categories	Subcategories	Main categories	Subcategories
Biological factors	Gender, Age, Physical activity level, Fatigue	Interaction with the care and treatment team	Quality of communication with physicians, quality of communication with nurses, quality of communication with other health team members, trust in health care providers, professional competence of health team members
Cognitive factors	Cognitive ability, problem-solving ability health literacy	Family connections	Family caregiver knowledge, Support from family members, Caregiver burden
Co-morbidities	Pain, frailty, severity of diseases, polypharmacy, complications of treatment	Facilities of medical centers	The existence of sufficient healthcare resources, Responsiveness of health services to the needs of the older adults, Well-equipped medical centers
Mental health	Anxiety, depression, loneliness, sleep issues, substance abuse, life satisfaction, sensitivity and perceived severity of disease complications, happiness, personality type	Empowering employees	Manpower training improving job satisfaction of employees
Economic-social factors	Cost of treatment and care, patient’s financial ability, formal social support structures	Health policymaking	Health system’s approach to older adults participation (patient-centered), treatment protocols, insurance coverage
Health-oriented behaviors	Adherence to treatment, healthy lifestyle	Cultural factors (cultural beliefs)	Altruism in society ageism, traditional religious beliefs about the older adults

In the category of biological factors, the analysis of integrated review data showed that gender (27, 38–42), age (27, 40–42) and physical activity level (17, 28, 43, 44) affect the self-management of older adults with MCCs. Additionally, participants’ experiences indicated the effect of fatigue on self-management. Regarding gender, the results of the studies were conflicting. However, most studies have shown greater self-management among older adult women (38, 45, 46). A nurse (P10) said: “*… older adult women have better control over their conditions and follow treatment plans independently and more regularly than older adult men*”.

In the category of cognitive factors, abilities such as cognitive ability, self-efficacy, self-care ability (3, 17, 24, 27–29, 40, 41, 44, 47–49), self-efficacy, self-care ability, self-management skills (24, 27, 50–52), and problem-solving ability (3, 28, 30, 40, 41, 47, 53) promote self-management and health literacy (17, 24, 28, 41, 46, 51, 54–59) which are directly related to self-management. Participants also confirmed these factors. A 48-year-old male nurse (P8) acknowledged that: “…*The older adult’s high level of education creates resistance to receiving effective care from formal caregivers. They search the internet, ask people, and want to do a better job*.” A Gerontology specialist (P3) added: “*… In old age, mental abilities decline leading to memory issues forgetfulness, and cognitive changes*”.

In the category of comorbid diseases, pain (17, 27, 42, 53, 56), the severity of diseases (25, 27, 29, 42, 46, 52, 56, 59), treatment complications (53, 56, 59–62), frailty (24, 41, 63) and polypharmacy (53, 56, 61) have been mentioned. A 37-year-old female caregiver (P18) said:”*…His illness puts a lot of pressure on him and it becomes severe. When we see my father’s pain and moans, we knock on every door to calm him. When he is in pain, we cannot even talk to him*.” A 41-year-old male nurse (P10) said: “…*Polypharmacy in older adults causes them to fall, get poisoned, and feel lethargic, and in this situation, they cannot take care of themselves and need support*”.

Economic-social factors, such as the cost of treatment and care (28–30, 41, 56, 64, 65), the patient’s financial ability (52, 55, 65–67), and formal social support structures (3, 28, 68–70) affect self-management. A 28-year-old caregiver (P19) expressed, “*…Grandma’s economic status is not high, and we have to pool money from family members to buy equipment or pay for doctor visits*.”Another geriatric specialist (P5) mentioned, “*… Older adults derive happiness from being active in the community, which in turn affects their self-management at an interpersonal level*.” The 28-year-old caregiver (P19) added, “*…The role of the older adults in our society has diminished, causing isolation and difficulties, especially when they fall ill*”.

Health-oriented behaviors encompass treatment adherence (44, 49, 71–73) and maintaining a healthy lifestyle (17, 28, 40, 49, 74, 75). A 73-year-old man (P20) shared, “… *Despite my health issues, I diligently followed my knee surgery plan from start to finish.*” An 81-year-old woman (P21) mentioned, “*… I visit the gym three times a week and engage in regular walks*.” A physiotherapist (P10) noted, “*… Efforts to support and care for the older adults play a significant role in maintaining their independence*.” A female nurse (P12) emphasized, “*… Consistency in following up on health recommendations is crucial for improving the quality of life among the older adults*”.

The mental health category included anxiety (17, 52, 57, 66), depression (17, 25, 57, 66), feelings of loneliness (17, 57), life satisfaction (17, 40, 41, 64, 76), sleep disorders (44, 74, 77), and substance abuse (20, 76). The participants highlighted the impact of these factors on self-management. A male psychologist (P1) stated that “*… Depression is common among older adults with chronic illnesses, leading to withdrawal and anxiety*.” A 65-year-old woman (P22) stated, “…*The first two years of my illness were challenging, and I felt nervous and depressed, and my condition worsened. I gradually accepted it*.” The caregiver of a 37-year-old woman (P18) shared, “…*Now, due to all the pain and suffering they endure, they are in denial and refuse treatment. If they continue to deny it, they will experience mental and physical complications.*” The *older adult* participants viewed happiness as a key factor in improving self-management, with an 81-year-old woman (P21) mentioning: *“…I make an effort to find happiness*.” A 67-year-old woman added: “…Happiness boosts my optimism and helps me cope with challenges”.

Interaction with the care and treatment team involves quality communication with physicians (78–80), nurses (68, 80), and other healthcare team members (81, 82), trust in healthcare providers (29, 38, 61, 76), and the professional competence of the healthcare team (40, 43, 64). The participants emphasized the importance of effective communication with healthcare professionals. One clinical psychologist (p1) said: *“… I prioritize clear communication and active listening when caring for older adult patients.”* A female caregiver (p14) highlighted: “… *The role of hospital follow-ups and trust in health care played a significant role in my mother’s well-being*”.

Family relationships involve family caregivers’ knowledge (3, 59, 61, 83); and support from family members (61, 68, 73, 84). The participants emphasized the role of family support in the self-management of older adults. The Faculty of Gerontology (P4) stated: “*…Many older adults people who receive sufficient support from their families prefer to carry out their daily activities independently as much as possible*.” A 48-year-old nurse (P8) said: “*…The demanding nature of caring for older adults can sometimes strain these relationships, causing them to deteriorate*.” (P7) said: “… *is a surprise that many older adults feel lonely and isolated. This issue has become increasingly prevalent in society*”.

Medical center facilities include limited access to specialized healthcare centers (24, 66), responsiveness of health services to older adults’ needs (69, 76, 85–88), and equipped medical centers (3, 59, 61). Participants stressed the importance of a responsive healthcare system for older adults. A geriatrics specialist (P7) said, “… *Now is the time when we need to establish special education systems for older adults and improve access to services; under the direct supervision and support of the Ministry of Health*.” The Faculty of Gerontology (P4) stated, “*…The crucial issue is the comprehensive care program itself, which is conducted by the Ministry of Health and Medicine. Proper screening, training, and media should also be implemented to avoid escalating concerns among older adults*.” A 28-year-old caregiver (P19) said, “*… There are no specialized sections for older adult patients, making it challenging for them and their families to navigate the situation*.” A physician specializing in geriatrics (P2) said, “*…Now, the best practice is to establish an integrated consultation system for older adults, offer follow-up services, and address the issue of worn-out equipment in medical centers*.” The 28-year-old caregiver (P19) said: “…*Let us establish specialized centers so that I do not have to worry so much about caring for an older adult person. It is unbelievable that we had to wait for hours before he could see the physician and visit the pharmacy*”.

Employee empowerment involves human resource training (30, 43, 89) and promoting employee job satisfaction (90–93). Participants emphasized the importance of job satisfaction and specialized care in improving self-management among older adults. A 36-year-old female caregiver (P15): “*…Those who are dissatisfied and have had a negative experience in their jobs may behave in ways that transmit these negative feelings to older adults, ultimately worsening their condition*.” A Female geriatric specialist stated (P5): “… *To provide high-quality follow-up care and improve job satisfaction, it is necessary to design a care quality monitoring system and a promotion system for geriatric specialist caregivers*”.

A 48-year-old female nurse (P12): “*…Working with older adults is not easy, especially for those with MCCs. Therefore. It is crucial to focus on specialized training in older adults care*”.

Health policy-making includes the health system’s approach to older adults participation (53, 69, 76, 85–87), treatment protocols (29, 40, 51, 65, 66), and insurance coverage of treatment costs (51, 88). The participants discussed the need for improved healthcare policies to improve the *older adult* population. A geriatric specialist (P4) said: “*…Our network system is highly efficient, but for a patient-centered approach, a specific set of facilities must be clearly defined at the center, aligned with the network system. However, access points are not properly defined*.” A 41-year-old male nurse (P10) said: “*…The older adult patients show improvement while in the hospital, they receive training, during their stay and continue to do so after discharge. However, the main issue with our caregivers is the lack of specific instructions for older adults, which results in a lack of follow-up and instances of neglect*.” A 46-year-old nurse (P8) said, “*…I always recommend having a nursing home for older adults in our country. This can promote teamwork, solidarity, empathy, and harmony among the older adults as well as a sense of vitality and well-being*.” A 45-year-old caregiver (P16) expressed concern, stating.”*…With each incident and government decision, access to medicine and health facilities is diminishing. Medicine is becoming increasingly scarce, and I am anxious about managing my mother’s illness*”.

Cultural factors include altruism in society (63, 94–97), discrimination and ageism (28, 40, 51, 57, 69, 80, 97, 98), and traditional-religious beliefs about *older adults* (57, 59, 99). The participants highlighted the influence of cultural factors on decision-making and self-management among older adults. A 48-year-old nurse (P8) said, “In our culture, we have a deep respect for *older adults*. It is important for them to make their own decisions and take care of themselves. We must assist them in any manner that they may be required. Let us make it happen.” The physiotherapist (P11) said, “*…The older adults possess valuable cultural capital and have developed a culture of effective self-care*.” The social worker (P9) admitted, “…When *older adults* are not a priority in the health system, this discrimination leads to their exclusion and a decrease in the quality of care in the future.” A 37-year-old female caregiver (P18), “…*The family’s attitude towards the older adults and recognizing their value is crucial; we must cultivate a culture that values the older*”.

## 4 Discussion

This qualitative study was conducted in Iran to identify the factors affecting self-management of older adults with MCCs. The findings revealed that self-management among *older adults* with MCCs was complex and interconnected. Each of these factors is discussed in this section. Gender is one of the biological variables associated with self-management. It was found that self-management tended to be higher in males (26). A study comparing self-management in women and men with MCCs; interpreted the structures of self-management as similar (55), although another study reported higher self-management among *older adult* women (46). Although men are more likely to receive self-management interventions, they often struggle with weaker self-management because of difficulties in seeking emotional support (38).

When self-management conflicts with the valuable aspects of men’s identity, especially their independence, they do not feel comfortable participating in self-management programs. However, when men understand support self-management, they provide and integrate it into their daily activities as are more likely to engage (38, 46). In addition to the contradictions in the findings, there is a need to improve self-management among older adult men and women (38, 45).

Self-management ability decreases with age, especially in older adults (41). The findings indicate that the level of daily activity plays a significant role in the self-management of older adults, this is supporting evidence suggesting that a decrease in activity and performance due to the older adults (42) leads to independent performance of daily tasks. Resulting in a decline in self-management. According to the experiences of the participants, older adults often feel tired. Older adults with MCCs who experience general fatigue tend to become more dependent on assistance with daily tasks, leading to a decrease in self-management (17, 28, 44).

Cognitive ability in older adults with MCCs is closely linked to self-management; therefore, managing cognitive symptoms in this population is crucial, as a poor understanding of preventive behaviors (17, 28, 40, 41), amnesia (24), memory loss (40), and dementia (27) can all contribute to a reduction in self-management. The findings emphasized that self-efficacy increases self-management; Söderlund et al. also showed that motivational components, including readiness for change, self-monitoring and goal setting, holding reinforcement meetings, feedback and problem-solving skills, self-regulation, and self-efficacy for problem-solving maintain self-management behavior. MCCs have been found in older adults (68).

Self-care ability is low in older adults with MCCs (28, 49) and self-management is high in older adults with independent self-care (27). Additionally, adherence to the self-care regime (3), sustainable behavioral changes (73), and promotion of empowerment are directly related to self-management (28), Accepting responsibility for self-care improves self-management and helps increase the quality of life of older adults (59). Chan et al.’s study has pointed to the direct relationship between empowerment and self-management (28) which helps increase the quality of life of the older adults (59). Self-monitoring of medications and conscientiousness (49) and self-management behaviors, such as proper diet, are related to self-management in older adults (72, 100).

Self-management skill is a challenge in older adults with chronic diseases, but its exact process has not been thoroughly investigated (52); about 30 self-management programs are mentioned in scientific sources, with 24 being private programs and six being public programs (89) Decreased self-management skills (51), rehabilitation (50), management of cognitive symptoms (28, 40, 41, 53), and a self-care regimen can reduce disability and improve the quality of life of older adults with MCCs (3). Older adults with MCCs often experience a decrease in self-control when it comes to diet, weight control, and exercise, which reduces self-management (72). Therefore, older adults must acquire self-management skills (28, 41). Improving problem-solving skills, health behaviors (17), and patient activity levels can increase self-management (74). Engaging in Positive health-oriented behaviors can enhance self-management (40). According to these findings, problem-solving ability is a significant factor in self-management. Evidence has shown that behaviors to adapt to disease (101), the ability to adapt to diseases (3), problem-solving skills (40, 41, 53, 101), and the belief that problem-solving ability decreases in older adults with MCCs (51) are all important factors.

low health literacy in older adults with MCCs is inversely related to self-management. Lack of knowledge about the disease leads to errors in acquiring self-management skills (60). Education has been shown to improve health treatment adherence and self-care among older adults (48, 54, 102). McGowan et al. have acknowledged that self-management training increases self-efficacy and improves treatment outcomes (56). The results highlight the importance of knowledge and awareness of self-management. However, effective knowledge and skills in caring for older adults with MCCs are limited (28). The lack of counseling and education is a significant barrier to self-management among older adults with chronic diseases (54, 55). Education (43), patient knowledge of disease management, and increased awareness (53, 67). Contribute to improved self-management, which in turn increases self-efficacy and enhances treatment outcomes (56).

Effective disease control knowledge and skills (28, 53), patient education (43, 48, 54), disease awareness (12, 31, 55–57), and health literacy levels (37); are crucial factors in promoting self-management. However, it is important to note that older adults can experience a decrease in information, potentially impacting self-management in older adults with MCCs. Therefore, health literacy education is important for patients with chronic diseases (46). Nguyen et al.’s study described reduced physical and cognitive functions; as well as health literacy (103). Byrne et al.’s research also highlighted the relationship between health literacy and mental health problems (104). Influencing the self-management of older adults with MCCs. Additionally, behavioral factors were found to have a direct impact on self-management, as demonstrated in Jarnet et al. study; on adherence to a self-care regime (73). McCabe et al., demonstrated a direct relationship between self-management and sustainable care behavior which is consistent, with previous research (44).

The findings highlight the significant role of mental health in self-management. This is particularly true for the older adult population. Mental health conditions in older adults, such as MCCs, are often associated with psychological issues (28); including increased anxiety, emotional distress (17, 66), and stress (57), which can act as barriers to effective self-management (46). Additionally, the time-consuming nature of self-management behaviors, coupled with negative emotions, fatigue, and threats to one’s identity, can impede successful self-management efforts (76).

Maintaining good mental health, coping with illness, engaging in social interactions, and communicating effectively with others can help increase participation in self-management programs (3). Sleep problems are indicated as a factor influencing self-management, as poor sleep quality can impact daily functioning (44, 77). Mental stability in older adults (74) helps increase the success of self-management programs. Self-management obstacles in older adults with MCCs are (17, 57, 86), low mood (57), and isolation and loneliness (17, 57). Older adults with mood disorders (71), anxiety, and depression (51, 71, 77) have lower self-management, and higher self-confidence is directly related to self-management (41). Decreased self-confidence and feelings of helplessness, pain, emotional distress, adaptability, management of cognitive symptoms (41, 51), and positive psychological attitude are directly related to self-management in older adults with chronic diseases (41). In the present study, substance abuse was considered to be an obstacle to effective self-management. In older adults, alcoholism, smoking, drug use, and excessive medication use hinder self-management (25, 44).

Poor understanding of preventive behaviors and negative beliefs about continuing life cause a decrease in self-management behaviors (73). Life satisfaction leads to higher self-management because suffering from MCCs is accompanied by a change in attitude towards life caused by diseases and the formation of beliefs related to diseases, which affect self-management. Stress management (52), attitudes and beliefs, disease experiences, and balance between life goals are related to self-management, and in older adults with chronic diseases, due to the time-consuming nature of self-management behaviors, such as negative, uncomfortable, boring, and sometimes threatening one’s identity are obstacles to successful self-management (40). Older adults with anxiety and depression (44, 105) have lower self-management, and higher self-confidence is directly related to self-management (53); therefore, it is suggested to strengthen positive psychological attitudes (40), which are directly related to self-management in older adults with chronic diseases (51). The findings showed that comorbid diseases has negative effects on self-management owing to complex treatment patterns. The need for re-rehospitalization, inappropriate response to treatment, the complexity of disease control and treatment, events and fluctuations in health status, and indicators of response to treatment (24) all have an impact on self-management, levels. Musculoskeletal pain is common among older adults and can hinder self-management as indicated by the findings of the present study (53).

Research has demonstrated that both acute and chronic pain in older adults can serve as predictors of self-management. Additionally reducing pain and disability can lead to an increase in self-management among older adults (27). Frailty in older adults with chronic diseases reduces their self-management abilities. Disease severity can also impact self-management, with symptoms ranging from moderate to severe outcomes, sudden relapse (32), disease progression, and high mortality risk (59) serving as predictors. Incurable diseases and the debilitating nature of chronic illnesses in older adults create challenges in disease control (21, 29). Treatment complications include disruptions to recommendations and difficulties in older adult patients agreeing to suggested treatments, which can hinder self-management and decision-making (28). Additionally, complications such as polypharmacy (59, 60), energy loss from drug consumption (59), and drug interactions (20) can further reduce self-management capabilities.

Socioeconomic factors also play a role in self-management (57) with high costs of living (75), economic restrictions (76, 77), factors such as low financial income (39), retirement, and inability to work (76, 77). Job changes can all negatively affect self-management in individuals with MCCs (56). Older adults with MCCs often face high medical expenses to maintain their health (71). Poleg et al. increased the frequency of physician visits, which directly correlated with higher treatment costs (59, 81, 106).

The promotion of self-management among older adults has been shown to reduce hospitalizations and treatment costs (18), as well as decrease emergency visits (20, 51). The key point is that individuals with chronic diseases have an increased need for home care services. Promoting self-management can help reduce the cost of care (59). Research has shown that an increase in treatment costs directly and indirectly affects self-management. Poleg et al. find that older adult with MCCs often faces high treatment costs to maintain their health (88).

Heid et al. and Chan et al. demonstrated that effective self-management can decrease the frequency of physician visits by up to 60%, especially in emergencies (29). Conversely, low levels of self-management can lead to higher treatment costs, as supported by studies conducted by Dye et al. (65), Ersek et al. (25), Elzen et al. (41), and Masters et al. (3). According to the findings, stable social support and support from friends had a statistically significant relationship with self-management. Reducing social activities (57, 67), and increasing the level of social interaction among older adults (56), and social participation (57) can impact self-management. Despite receiving disproportionate services, older adults strive to maintain their health and may face problems and needs related to the disease (54). Older adults with MCCs who have a higher quality of life tend to have better physical and mental functioning and self-management (55).

Adherence to medication regimens, self-monitoring of medications, and attention to treatment regimens for chronic diseases, can increase self-management in older adults with MCCs. Noncompliance with dietary recommendations is often linked to lower levels of self-management (51, 71), highlighting the importance of adhering to treatment regimens for chronic diseases related to self-management. Engaging in exercise (55). Maintaining an active lifestyle (55), adopting healthy behaviors (78), taking responsibility (29), making sustainable health behavior changes, being conscientious (78), enhancing adaptation skills, and adjusting lifestyle choices can all contribute to achieving self-management (40, 52) and finding a balance between self-management goals and life goals. The quality of communication with the treatment team is a factor related to treatment planning, follow-up care, and increasing self-management (106). Proper communication with specialists plays an effective role in implementing self-management programs, increasing the satisfaction of older adults, and improving their self-management (17, 67, 82).

Favorable therapeutic communication with physicians and specialists (17) increases self-management, whereas low levels of counseling and poor communication with physicians (17, 25, 57) decrease self-management. A positive therapeutic relationship between the physician and patient has a significant effect on self-efficacy and behavioral outcomes in people with chronic diseases (55); the quality of communication with other members of the treatment team including increasing support from the health team, and communication training with the health team (81), enhances self-management, and Poor communication with other treatment personnel, such as physiotherapists, psychologists, and occupational therapists, acts as an obstacle to self-management (82).

Poor communication with nurses (80, 82) and unfavorable team participation (57) had negative effects on the self-management outcomes of older adults with MCC. The optimal support provided by nurses for older adults and their families (28, 68) is directly related to self-management. Older adults trust formal caregivers and health service providers (76) along with a sense of security (51) ensures improved self-management. Additionally, a low level of trust prevents older adults from accepting the proposed treatment (73, 97). Dongbo et al. showed that increasing the support of health service providers improves the health status of older adults with MCC and reduces their disability (53). Sunert et al. showed that the initial trust of older adults in health service providers is related to the promotion of self-management (76). The findings highlight the impact of professional competence among health team members on self-management. Skilled personnel can assist older adults in better adaptation, diagnosing problems and educational needs (40, 64), planning and decision-making based on the situation of older adults, and counseling (28, 53), all of which contribute to improving self-management. Conversely, the lack of knowledge to enhance care and the failure of personnel to utilize specialized knowledge of geriatrics (30, 43) are negative factors that hinder self-management. Therefore, health and treatment systems require a proficient workforce to address these challenges.

Family caregivers’ skills in chronic disease management (83); and the conscious participation of family members in care (3, 61) increase self-management. Older adults living alone (57) and those with low family support (73) have decreased self-management. Effective communication with a spouse increases self-management, and proper adaptation to the disease improves self-efficacy. The family’s assurance of proper care (30), mutual support among family members and older adults (28, 68), age of the caregiver, and family participation are important in the self-management program for older adults (49). The results showed that family support and communication with friends and medical personnel are directly related to self-management. Masters et al. acknowledged that effective communication with spouses and family support increases self-management, promotes proper adaptation to the disease, and enhance self-efficacy. Caregiver age and family involvement are important factors in older adult self-management programs (3). Barriers to accessing resources, services, and specialized healthcare centers (48, 61), as well as difficulties in contacting service providers (61), hinder the utilization of beneficial treatment and impede self-management (48).

Providing telephone answering services for older adults (61), the successful use of patient-specific programs (76), successful implementation of patient-specific programs, and tailoring standard care programs to meet the needs of older adults with MCCs are feasible and cost-effective solutions (61). This cost-effectiveness will not only enhance self-management but also help control the risk factors associated with chronic diseases. Additionally, targeted monitoring of effective care (53), telephone monitoring of care programs for older adults (85), implementation of follow-up care programs at treatment centers for older adulthood (69, 87), and the development and strengthening of care services for older adults (79) are essential components in establishing a comprehensive care system. By focusing on chronic diseases and promoting a team approach to comprehensive care (88), a suitable platform was provided for the successful implementation of self-management programs. The use of information technology centers in controlling caring for; and treating chronic diseases, as well as utilizing sensor devices in web-based self-monitoring of care programs and developing technology centers to serve older adults (85), all play a crucial role in facilitating the diagnosis, treatment, and care of older adults affected by MCCs, ultimately enhancing the quality of their care. Reducing the budget for the purchase of equipment for equipment purchase relationship centers for older adults and the lack of diagnostic and laboratory facilities (88) indirectly impacts the self-management abilities of older adults.

Based on these findings, empowering care workers for older adults is crucial for promoting self-management. One obstacle to promoting self-management among older adults with MCCs is the shortage of human resources and insufficiently trained personnel. This results in limited educational opportunities (30, 89). MCCs are characterized by a shortage of human resources and insufficiently trained personnel. This results in limited educational opportunities (43, 61, 102), regular visits (30), effective communication, and the implementation of special self-management programs for older adults (17, 30). Therefore, it is essential to train specialized personnel in geriatrics, improve the skills of employees the skills of employees in older adult care, and utilize modern methods to train geriatric specialists (89). The job satisfaction of personnel is directly related to the promotion of self-management empowerment among older adulthood and the overall improvement in the quality of care. Management structures that provide a promotion system for older adult care personnel and their participation in decision-making have been found to increase self-management (93).

The study also highlighted the importance of health policy-making and the health system’s approach to involving older adults in decision-making at the national level. There is a growing need for a comprehensive and integrated care system as well as a systemic approach to providing comprehensive care (88). To effectively implement programs for older adults’ participation in care (76), it is essential to establish a standard care program and focus on older adults with MCCs within healthcare organizations. Innovation in monitoring and providing necessary care for older adults is also crucial (76, 86). Current treatment guidelines in this area are inadequate and action in national policy-making is necessary (76). policy-makers should be educated on the importance of self-management for chronic patients (85), standardized care levels (40, 51), upgrading facility resources (66, 86), and ensuring sustainable financial support for the older adults support system (29, 65) have also been recommended. Implementing a regular monitoring approach during patient care can empower patients by increasing their knowledge of their existing conditions; and enhancing their interactions with healthcare providers. Organizational supervision provides a suitable platform for the successful implementation of self-management programs. Studies by Liu et al. and Söderlund et al. showed that the lack of regular follow-up care and treatment (68, 97) is a significant obstacle to promoting self-management. Baylis et al. identified limited access to healthcare resources (66) while Joo et al. highlighted confusion in contacting service providers as an important barrier to implementing self-management among older adults with chronic diseases (24). Additionally, Liu et al. demonstrated that complications of that treatment complications can lead to disruptions in recommendations and difficulties in agreeing with proposed treatment plans. Self-management strategies and decision-making processes for older adults with chronic diseases (97).

Caring for older adults with MCCs poses a challenge to policymakers. Limited resources, a lack of specialized human resources, insurance issues, and restricted access to healthcare services are some of the primary obstacles in the field of policymaking. Jarnet et al. highlighted the lack of insurance coverage (73), while Boyd et al. point out that the absence of formal training programs for personnel and low levels of access to medical and care services as factors hindering self-management in older adults with MCC (79). This study also revealed that cultural factors play a significant role in the self-management of older adults. Older adults are viewed as cultural assets (95) and use media platforms to promote their dignity (76), thus supporting the development of cultural services and bases for older adult’s care (95).

Embracing a positive cultural perspective on caring for older adults (97) emerged as a crucial predictor of self-management in this demographic. Ultimately, there are positive cultural beliefs. Values surrounding older adults’ care greatly (97) influence self-management practices. A positive attitude towards older adults reduces the impact of diseases and promotes self-management. Conversely, a negative attitude towards older adult care leads to a decline in the quality of care, decreased motivation for treatment, and increased mortality rates (98). Indifference towards older adults (53, 69) hinders self-management while insulting behaviors and negative attitudes prevent participation in decision-making. Lower self-esteem and impeded effective self-management; marginalization and rejection reduces social roles (80) and self-management. Chan et al. (28) highlight the negative effects of rejection and marginalization on self-management and social isolation. Richardson et al.’s findings indicate a decrease in family roles and self-management among older adults (80).

Cultural beliefs play a significant role in older adults. Various organizations, including municipalities, utilize the capacities of the media to promote the dignity of older adults (76) and support the development of cultural services for them (95). Retirees should consider participating in cultural and social programs. Traditional approaches to health, viewing caring for older adults as a natural family responsibility, and relatives’ adherence to traditions and beliefs are closely linked to self-management in older adults with MCCs. The study’s findings suggest that altruism within society is connected to the enhancement of self-management skills. A culture of altruism in society can increase dignity, adaptability, and self-management (63, 94, 95, 97).

Delphi’s findings confirmed a set of factors related to self-management that resulted from an integrated literature review and qualitative interviews and added four additional variables: individual knowledge, cost of care, complex treatment patterns, use of self-management program due to integration with other variables, and disease severity.

### 4.1 Limitations

One limitation of the current study was the language restriction in selecting primary studies, which was confined to the English and Persian languages. However, this study has several strengths, such as a specific study design that integrates existing quantitative and qualitative evidence of the incorporation of experts, older adults, and family caregiver experience, and the use of the Delphi technique, which enhances the credibility of the study. It also considered various aspects to understand the multidimensional phenomenon of self-management comprehensively.

## 5 Conclusion

Self-management is considered a cornerstone of chronic disease care and the initial step in providing patients with the information they need to care for their health. MCCs’ self-management has been defined as the ongoing process of facilitating the knowledge, skill, and ability necessary for MCCs’ self-care. This process incorporates the needs, goals, and life experiences of a person with MCCs, and is guided by empirical evidence. The overall objectives of self-management are to support informed decision-making, self-care behaviors, problem-solving, and active collaboration with the healthcare team; and to improve clinical outcomes, health status, and quality of life.

The findings indicate that the level of self-management in older adults with MCCs is unfavorable. Given the growing older adult population, it is crucial to identify factors related to self-management to develop specialized models and interventions to maintain and improve health. Self-management in older adults is influenced by various factors, including older adults, their families, caregivers, disease complications, the healthcare system, society, culture, and politics. Therefore, a comprehensive approach is necessary when designing interventions and services for this population. It is expected that the application of the findings of this research in the clinical and policy-making of health systems will have positive consequences. According to the present results, a future line of research can expand the sample to include older adulthood population suffering from various diseases and chronic syndromes. The expansion of study variables that can predict self-management and may not be considered in this study should be considered in future studies. It is also recommended that the evaluation of some variables related to self-management, such as frailty, should be performed through biometric measurement methods and not only through self-administered questionnaires.

The theoretical implications of this study will help strengthen the existing primary scientific evidence on physical, cultural, psychological, and social predictors of self-management in older adulthood with MCCs because the results of this project are the most up-to-date local evidence on variables related to self-management in older adulthood. In this regard, this project can help to address issues related to the production of a self-management predictive model of older adulthood with MCCs visible in the scientific community. In addition, the results of this study may help Geriatric specialists to better diagnose the factors affecting the self-management of older adulthood under treatment and care and help improve the quality of life of older adulthood.

In terms of practical implications, these results can serve as evidence for geriatric health decision-makers in developing clinical programs to examine predictors of self-management. On the other hand, it is possible to propose strategies to promote healthy aging based on the promotion of variables that are recognized as predictors of low self-management. In particular, the study methodology can be repeated, and as a result, these results can be confirmed in other older adult populations, in other environments with different barriers and facilitators of self-management, and in improving prevention and intervention variables. The strengths of this study, in addition to the fact that it was conducted for the first time in Iran, is its multi-stage methodology.

## Data Availability

The original contributions presented in the study are included in the article/supplementary material, further inquiries can be directed to the corresponding authors.

## References

[1] Shen D Zhang M Bhandari B Yu D. Food additives manufacturing processing for elderly: advancements, issues, prospective solutions, and future direction. Food Bioprocess Technol. (2024) 2024:1–19. doi: 10.1007/s11947-024-03331-1

[2] Pouladi S Anoosheh M Kazemnejad A Zareiyan A. Factors limiting families in elderly care: a thematic analysis. J Qual Res Health Sci. (2013) 2:146–57. [In Persian].

[3] Masters S Oliver-Baxter J Barton C Summers M Howard S Roeger L. Programmes to support chronic disease self-management: should we be concerned about the impact on spouses? Health Soc Care Community. (2013) 21:315–26. doi: 10.1111/hsc.12020, PMID: 23441887

[4] Doshmangir L Khabiri R Gordeev VS. Policies to address the impact of an ageing population in Iran. Lancet. (2023) 401:1078. doi: 10.1016/S0140-6736(23)00179-4 37003695

[5] Goharinezhad S Maleki M Baradaran HR Ravaghi H. Futures of elderly care in Iran: a protocol with scenario approach. Med J Islam Repub Iran. (2016) 30:416. 28210581 PMC5307636

[6] Bauer UE Briss PA Goodman RA Bowman BA. Prevention of chronic disease in the 21st century: elimination of the leading preventable causes of premature death and disability in the USA. Lancet. (2014) 384:45–52. doi: 10.1016/S0140-6736(14)60648-6 24996589

[7] Hossain ME Khan A Uddin S. *Understanding the comorbidity of multiple chronic diseases using a network approach*. Proceedings of the Australasian Computer Science Week Multiconference (2019).

[8] Newman D Levine E Kishore SP. Prevalence of multiple chronic conditions in New York state, 2011–2016. PLoS One. (2019) 14:e0211965. doi: 10.1371/journal.pone.0211965 30730970 PMC6366719

[9] Ahmadi B Alimohammadian M Yaseri M Majidi A Boreiri M Islami F. Multimorbidity: epidemiology and risk factors in the Golestan cohort study, Iran: a cross-sectional analysis. Medicine. (2016) 95:e2756. doi: 10.1097/MD.0000000000002756, PMID: 26886618 PMC4998618

[10] Nejati V Shirinbayan P Akbari Kamrani A Foroughan M Taheri P Sheikhvatan M. Quality of life in elderly people in Kashan, Iran. Middle East J Age Ageing. (2008) 5:21–5.

[11] Yu T Enkh-Amgalan N Zorigt G Hsu Y-J Chen H-J Yang H-Y. Gender differences and burden of chronic conditions: impact on quality of life among the elderly in Taiwan. Aging Clin Exp Res. (2019) 31:1625–33. doi: 10.1007/s40520-018-1099-2, PMID: 30604210

[12] N’Goran AA Déruaz-Luyet A Haller DM Zeller A Rosemann T Streit S. Comparing the self-perceived quality of life of multimorbid patients and the general population using the EQ-5D-3L. PLoS One. (2017) 12:e0188499. doi: 10.1371/journal.pone.0188499, PMID: 29261695 PMC5736180

[13] Voetsch K Sequeira S Chavez AH. Peer reviewed: a customizable model for chronic disease coordination: lessons learned from the coordinated chronic disease program. Prev Chronic Dis. (2016) 13:E43. doi: 10.5888/pcd13.150509, PMID: 27032986 PMC4825748

[14] Parra-Rizo MA Díaz-Toro F Hadrya F Pavón-León P Cigarroa I. Association of co-living and age on the type of sports practiced by older people. Sports. (2022) 10:200. doi: 10.3390/sports10120200 36548497 PMC9785896

[15] Agustí AI Guillem-Saiz J González-Moreno J Cantero-García M Cigarroa I Parra-Rizo MA. Predictors of health satisfaction in Spanish physically active older adults: a cross-sectional observational study. Geriatrics. (2023) 8:27. doi: 10.3390/geriatrics8010027, PMID: 36826369 PMC9957470

[16] Vos T Flaxman AD Naghavi M Lozano R Michaud C Ezzati M. Years lived with disability (YLDs) for 1160 sequelae of 289 diseases and injuries 1990–2010: a systematic analysis for the global burden of disease study 2010. Lancet. (2012) 380:2163–96. doi: 10.1016/S0140-6736(12)61729-2, PMID: 23245607 PMC6350784

[17] Warner G Packer TL Kervin E Sibbald K Audulv Å. A systematic review examining whether community-based self-management programs for older adults with chronic conditions actively engage participants and teach them patient-oriented self-management strategies. Patient Educ Couns. (2019) 102:2162–82. doi: 10.1016/j.pec.2019.07.002, PMID: 31301922

[18] Mackey LM Doody C Werner EL Fullen B. Self-management skills in chronic disease management: what role does health literacy have? Med Decis Mak. (2016) 36:741–59. doi: 10.1177/0272989X16638330 27053527

[19] Du S Yuan C Xiao X Chu J Qiu Y Qian H. Self-management programs for chronic musculoskeletal pain conditions: a systematic review and meta-analysis. Patient Educ Couns. (2011) 85:e299–310. doi: 10.1016/j.pec.2011.02.021 21458196

[20] Lukaschek K Schneider N Schelle M Kirk UB Eriksson T Kunnamo I. Applicability of motivational interviewing for chronic disease management in primary care following a web-based e-learning course: cross-sectional study. JMIR Ment Health. (2019) 6:e12540. doi: 10.2196/12540, PMID: 31033446 PMC6658265

[21] Aarts JW Deckx L van Abbema DL Tjan-Heijnen VC van den Akker M Buntinx F. The relation between depression, coping and health locus of control: differences between older and younger patients, with and without cancer. Psycho-Oncology. (2015) 24:950–7. doi: 10.1002/pon.3748 25644618

[22] Kahe M Vameghi R Foroughan M Bakhshi E Bakhtyari V. The relationships between self-concept and self-efficacy with self-management among elderly of sanatoriums in Tehran. Iran J Ageing. (2018) 13:28–37. [In Persian].

[23] Gholi Zadeh S Khankeh HR Mohammadi F. The effect of book therapy on elderly self-management capabilities. Iran J Ageing. (2012) 6:51–7. [In Persian].

[24] Joo J Lee H. Barriers to and facilitators of diabetes self-management with elderly Korean-American immigrants. Int Nurs Rev. (2016) 63:277–84. doi: 10.1111/inr.12260 26970224

[25] Ersek M Turner JA McCurry SM Gibbons L Kraybill BM. Efficacy of a self-management group intervention for elderly persons with chronic pain. Clin J Pain. (2003) 19:156–67. doi: 10.1097/00002508-200305000-00003 12792554

[26] Wang XL Cheng J Guo CY Xu XR. The implications of childcare on grandparents’ health self-management in a Chinese elderly population. Int J Health Plann Manag. (2020) 35:280–9. doi: 10.1002/hpm.2904 31657493

[27] Rios JCS Hua FY Safons MP. Posture-focused self-management programme improves pain and function in older people with chronic low back pain: a randomised controlled trial. Int J Ther Rehabil. (2020) 27:1–10. doi: 10.12968/ijtr.2018.0082

[28] ChanWLHuiEChanCCheungDWongSWongR. Evaluation of chronic disease self-management programme (CDSMP) for older adults in Hong Kong. J Nutr Health Aging. (2011) 15:209–14. doi: 10.1007/s12603-010-0257-921369669

[29] Heid AR Gerber AR Kim DS Gillen S Schug S Pruchno R. Timing of onset and self-management of multiple chronic conditions: a qualitative examination taking a lifespan perspective. Chronic Illn. (2020) 16:173–89. doi: 10.1177/1742395318792066, PMID: 30180778

[30] Harvey PW Petkov J Kowanko I Helps Y Battersby M. Chronic condition management and self-management in aboriginal communities in South Australia: outcomes of a longitudinal study. Aust Health Rev. (2013) 37:246–50. doi: 10.1071/AH12165, PMID: 23369208

[31] Whittemore R Knafl K. The integrative review: updated methodology. J Adv Nurs. (2005) 52:546–53. doi: 10.1111/j.1365-2648.2005.03621.x 16268861

[32] Nightingale R McHugh G Kirk S Swallow V. Supporting children and young people to assume responsibility from their parents for the self-management of their long-term condition: an integrative review. Child Care Health Dev. (2019) 45:175–88. doi: 10.1111/cch.12645 30690751

[33] Bakhtiyari F Foroughan M Fakhrzadeh H Nazari N Najafi B Alizadeh M. Validation of the Persian version of abbreviated mental test (AMT) in elderly residents of Kahrizak charity foundation. Iran J Diabetes Metab. (2014) 13:487–94. [In Persian].

[34] Kyngäs H Kääriäinen M Elo S. The trustworthiness of content analysis. Appl Content Anal Nurs Sci Res. (2020) 1:41–8. doi: 10.1007/978-3-030-30199-6_5

[35] Polit D Beck C. Essentials of nursing research: Appraising evidence for nursing practice. Philadelphia, PA: Lippincott Williams and Wilkins (2020).

[36] Guba EG Lincoln YS. Competing paradigms in qualitative research In: Guba EG, editor. Handbook of Qualitative Research, vol. 2. Thousand Oaks, CA: SAGE (1994). 105.

[37] Bergin T. An introduction to data analysis: quantitative, qualitative and mixed methods. Los Angeles: SAGE, pp. 1–296. (2018).

[38] Galdas P Darwin Z Kidd L Blickem C McPherson K Hunt K. The accessibility and acceptability of self-management support interventions for men with long term conditions: a systematic review and meta-synthesis of qualitative studies. BMC Public Health. (2014) 14:1–20. doi: 10.1186/1471-2458-14-1230 25428230 PMC4295235

[39] Roberto KA Gigliotti CM Husser EK. Older women’s experiences with multiple health conditions: daily challenges and care practices. Health Care Women Int. (2005) 26:672–92. doi: 10.1080/07399330500177147, PMID: 16234211

[40] Smeulders ES Van Haastregt JC Ambergen T Uszko-Lencer NH Janssen-Boyne JJ Gorgels AP. Nurse-led self-management group programme for patients with congestive heart failure: randomized controlled trial. J Adv Nurs. (2010) 66:1487–99. doi: 10.1111/j.1365-2648.2010.05318.x, PMID: 20492026

[41] Elzen H Slaets JP Snijders TA Steverink N. Evaluation of the chronic disease self-management program (CDSMP) among chronically ill older people in the Netherlands. Soc Sci Med. (2007) 64:1832–41. doi: 10.1016/j.socscimed.2007.02.008 17355901

[42] Convery E Meyer C Keidser G Hickson L. Assessing hearing loss self-management in older adults. Int J Audiol. (2018) 57:313–20. doi: 10.1080/14992027.2017.1390268 29081257

[43] Kelly C Grundy S Lynes D Evans DJ Gudur S Milan SJ. Self-management for bronchiectasis. Cochrane Database Syst Rev. (2018) 2018:12528. doi: 10.1002/14651858.CD012528.pub2 PMC6491127 29411860

[44] McCabe C McCann M Brady AM. Computer and mobile technology interventions for self-management in chronic obstructive pulmonary disease. Cochrane Database Syst Rev. (2017) 2020:11425. doi: 10.1002/14651858.CD011425.pub2 PMC6481891 28535331

[45] Pati S van den Akker M Schellevis FG Sahoo KC Burgers JS. Management of diabetes patients with comorbidity in primary care: a mixed-method study in Odisha, India. Fam Pract. (2023) 40:714–21. doi: 10.1093/fampra/cmac144 36610706

[46] Kroon FP van der Burg LR Buchbinder R Osborne RH Johnston RV Pitt V. Self-management education programmes for osteoarthritis. Cochrane Database Syst Rev. (2014) 1:CD008963. doi: 10.1002/14651858.CD008963.pub2 PMC11104559 24425500

[47] Babamohamadi H Tafreshi A Khoshbakht S Ghorbani R Asgari MR. Nursing students’ professional competence in providing spiritual Care in Iran. J Relig Health. (2022) 61:1831–47. doi: 10.1007/s10943-021-01365-9 34333688

[48] Zhang R Lu Y Shi L Zhang S Chang F. Prevalence and patterns of multimorbidity among the elderly in China: a cross-sectional study using national survey data. BMJ Open. (2019) 9:e024268. doi: 10.1136/bmjopen-2018-024268 PMC6701688 31427309

[49] Al-Rousan T Pesantes MA Dadabhai S Kandula NR Huffman MD Miranda JJ. Patients’ perceptions of self-management of high blood pressure in three low-and middle-income countries: findings from the BPMONITOR study. Glob Health Epidemiol Genom. (2020) 5:e4. doi: 10.1017/gheg.2020.5, PMID: 32742666 PMC7372177

[50] Brennan BA. The impact of self-efficacy based prebriefing on nursing student clinical competency and self-efficacy in simulation: an experimental study. Nurse Educ Today. (2022) 109:105260. doi: 10.1016/j.nedt.2021.105260, PMID: 34973554

[51] Reed RL Battersby M Osborne RH Bond MJ Howard SL Roeger L. Protocol for a randomised controlled trial of chronic disease self-management support for older Australians with multiple chronic diseases. Contemp Clin Trials. (2011) 32:946–52. doi: 10.1016/j.cct.2011.08.001 21864719

[52] O’Conor R Martynenko M Gagnon M Hauser D Young E Lurio J. A qualitative investigation of the impact of asthma and self-management strategies among older adults. J Asthma. (2017) 54:39–45. doi: 10.1080/02770903.2016.1193602, PMID: 27315570

[53] Dongbo F Ding Y McGowan P Fu H. Qualitative evaluation of chronic disease self management program (CDSMP) in Shanghai. Patient Educ Couns. (2006) 61:389–96. doi: 10.1016/j.pec.2005.05.002 15975756

[54] Werfalli M Murphy K Kalula S Levitt N. Current policies and practices for the provision of diabetes care and self-management support programmes for older South Africans. Afr J Prim Health Care Fam Med. (2019) 11:e1–e12. doi: 10.4102/phcfm.v11i1.2053, PMID: 31478747 PMC6739530

[55] Smith D Fairweather-Schmidt AK Harvey P Bowden J Lawn S Battersby M. Does the Partners in Health scale allow meaningful comparisons of chronic condition self-management between men and women? Testing measurement invariance. J Adv Nurs. (2019) 75:3126–37. doi: 10.1111/jan.14124 31236969

[56] McGowan PT. Self-management education and support in chronic disease management. Prim Care. (2012) 39:307–25. doi: 10.1016/j.pop.2012.03.005 22608868

[57] Stein-Parbury J Gallagher R Chenoweth L Luscombe G. Factors associated with good self-management in older adults with a schizophrenic disorder compared with older adults with physical illnesses. J Psychiatr Ment Health Nurs. (2012) 19:146–53. doi: 10.1111/j.1365-2850.2011.01767.x 22070648

[58] Dickinson WP Dickinson LM Jortberg BT Hessler DM Fernald DH Cuffney M. A cluster randomized trial comparing strategies for translating self-management support into primary care practices. J. Am. Board Fam. Med. (2019) 32:341–52. doi: 10.3122/jabfm.2019.03.180254, PMID: 31068398 PMC6599532

[59] Kawi J. Chronic low back pain patients’ perceptions on self-management, self-management support, and functional ability. Pain Manag Nurs. (2014) 15:258–64. doi: 10.1016/j.pmn.2012.09.003 23232149

[60] Rahman FI Aziz F Huque S Ether SA. Medication understanding and health literacy among patients with multiple chronic conditions: a study conducted in Bangladesh. J Public Health Res. (2020) 9:1792. doi: 10.4081/jphr.2020.1792 PMC7315107 32607317

[61] Dineen-Griffin S Garcia-Cardenas V Williams K Benrimoj SI. Helping patients help themselves: a systematic review of self-management support strategies in primary health care practice. PLoS One. (2019) 14:e0220116. doi: 10.1371/journal.pone.0220116 31369582 PMC6675068

[62] Tan SS Pisano MM Boone AL Baker G Pers Y-M Pilotto A. Evaluation design of EFFICHRONIC: the chronic disease self-management programme (CDSMP) intervention for citizens with a low socioeconomic position. Int J Environ Res Public Health. (2019) 16:1883. doi: 10.3390/ijerph16111883, PMID: 31142017 PMC6603786

[63] Keidar D Yagoda A. Emotional intelligence, moral, ethics, bio-ethics and what is in between. Med Law. (2014) 33:131. 27359022

[64] Bauman AE Craig AR Dunsmore J Browne G Allen DH Vandenberg R. Removing barriers to effective self-management of asthma. Patient Educ Couns. (1989) 14:217–26. doi: 10.1016/0738-3991(89)90034-7

[65] Dye C Willoughby D Aybar-Damali B Grady C Oran R Knudson A. Improving chronic disease self-management by older home health patients through community health coaching. Int J Environ Res Public Health. (2018) 15:660. doi: 10.3390/ijerph15040660 29614803 PMC5923702

[66] Bayliss EA Ellis JL Steiner JF. Barriers to self-management and quality-of-life outcomes in seniors with multimorbidities. Ann Fam Med. (2007) 5:395–402. doi: 10.1370/afm.722 17893380 PMC2000313

[67] Garnett A Ploeg J Markle-Reid M Strachan PH. Self-management of multiple chronic conditions by community-dwelling older adults: a concept analysis. SAGE Open Nurs. (2018) 4:237796081775247. doi: 10.1177/2377960817752471 PMC7774451 33415188

[68] Söderlund A von Heideken WP. Adherence to and the maintenance of self-management behaviour in older people with musculoskeletal pain—a scoping review and theoretical models. J Clin Med. (2021) 10:303. doi: 10.3390/jcm10020303, PMID: 33467552 PMC7830780

[69] Holmes MM Stanescu S Bishop FL. The use of measurement systems to support patient self-management of long-term conditions: an overview of opportunities and challenges. Patient Relat Outcome Meas. (2019) 10:385. doi: 10.2147/PROM.S178488 31908555 PMC6924578

[70] Wang C Pu R Li Z Ji L Li X Ghose B. Subjective health and quality of life among elderly people living with chronic multimorbidity and difficulty in activities of daily living in rural South Africa. Clin Interv Aging. (2019) 14:1285–96. doi: 10.2147/CIA.S205734, PMID: 31409978 PMC6645605

[71] Fryer CE Luker JA McDonnell MN Hillier SL. Self management programmes for quality of life in people with stroke. Cochrane Database Syst Rev. (2016) 2019:442. doi: 10.1002/14651858.CD010442.pub2 PMC6450423 27545611

[72] Zhang X Chen H Liu Y Yang B. Influence of chronic illness resources on self-management and the mediating effect of patient activation among patients with coronary heart disease. Nurs Open. (2021) 8:3181–9. doi: 10.1002/nop2.1031 34498405 PMC8510723

[73] Jerant AF von Friederichs-Fitzwater MM Moore M. Patients’ perceived barriers to active self-management of chronic conditions. Patient Educ Couns. (2005) 57:300–7. doi: 10.1016/j.pec.2004.08.004 15893212

[74] Van Puffelen AL Rijken M Heijmans MJ Nijpels G Schellevis FG Group DS. Effectiveness of a self-management support program for type 2 diabetes patients in the first years of illness: results from a randomized controlled trial. PLoS One. (2019) 14:e0218242. doi: 10.1371/journal.pone.0218242 31247039 PMC6597059

[75] Schulman-Green D Jaser S Martin F Alonzo A Grey M McCorkle R. Processes of self-management in chronic illness. J Nurs Scholarsh. (2012) 44:136–44. doi: 10.1111/j.1547-5069.2012.01444.x, PMID: 22551013 PMC3366425

[76] Sunaert P Vandekerckhove M Bastiaens H Feyen L Vanden Bussche P De Maeseneer J. Why do GPs hesitate to refer diabetes patients to a self-management education program: a qualitative study. BMC Fam Pract. (2011) 12:1–11. doi: 10.1186/1471-2296-12-94 21902832 PMC3181200

[77] Nicholas MK Asghari A Blyth FM Wood BM Murray R McCabe R. Self-management intervention for chronic pain in older adults: a randomised controlled trial. Pain. (2013) 154:824–35. doi: 10.1016/j.pain.2013.02.009 23522927

[78] Ory MG Ahn S Jiang L Lorig K Ritter P Laurent DD. National study of chronic disease self-management: six-month outcome findings. J Aging Health. (2013) 25:1258–74. doi: 10.1177/0898264313502531 24029414

[79] Boyd C Smith CD Masoudi FA Blaum CS Dodson JA Green AR. Decision making for older adults with multiple chronic conditions: executive summary for the American Geriatrics Society guiding principles on the care of older adults with multimorbidity. J Am Geriatr Soc. (2019) 67:665–73. doi: 10.1111/jgs.15809 30663782

[80] Richardson J Loyola-Sanchez A Sinclair S Harris J Letts L MacIntyre NJ. Self-management interventions for chronic disease: a systematic scoping review. Clin Rehabil. (2014) 28:1067–77. doi: 10.1177/0269215514532478 24784031

[81] Beattie JBM Pols RG. The acceptability and outcomes of a peer-and health-professional-led Stanford self-management program for Vietnam veterans with alcohol misuse and their partners. Psychiatr Rehabil J. (2013) 36:306–13. doi: 10.1037/prj0000031, PMID: 24219770

[82] Kastner M Hayden L Wong G Lai Y Makarski J Treister V. Underlying mechanisms of complex interventions addressing the care of older adults with multimorbidity: a realist review. BMJ Open. (2019) 9:e025009. doi: 10.1136/bmjopen-2018-025009, PMID: 30948577 PMC6500199

[83] De Bruin SR Versnel N Lemmens LC Molema CC Schellevis FG Nijpels G. Comprehensive care programs for patients with multiple chronic conditions: a systematic literature review. Health Policy. (2012) 107:108–45. doi: 10.1016/j.healthpol.2012.06.006, PMID: 22884086

[84] Cramm JM Twisk J Nieboer AP. Self-management abilities and frailty are important for healthy aging among community-dwelling older people; a cross-sectional study. BMC Geriatr. (2014) 14:1–5. doi: 10.1186/1471-2318-14-28 24602327 PMC3975729

[85] Jiang J Cameron A-F. IT-enabled self-monitoring for chronic disease self-management: an interdisciplinary review. MIS Q. (2020) 44:451–508. doi: 10.25300/MISQ/2020/15108

[86] Jobst S Leppla L Köberich S. A self-management support intervention for patients with atrial fibrillation: a randomized controlled pilot trial. Pilot Feasibility Stud. (2020) 6:1–18. doi: 10.1186/s40814-020-00624-y 32566244 PMC7301515

[87] Du S Liu W Cai S Hu Y Dong J. The efficacy of e-health in the self-management of chronic low back pain: a meta analysis. Int J Nurs Stud. (2020) 106:103507. doi: 10.1016/j.ijnurstu.2019.103507 32320936

[88] Ploeg J Yous M-L Fraser K Dufour S Baird LG Kaasalainen S. Healthcare providers’ experiences in supporting community-living older adults to manage multiple chronic conditions: a qualitative study. BMC Geriatr. (2019) 19:1–14. doi: 10.1186/s12877-019-1345-2 31744477 PMC6862842

[89] Jans G Lenzen S Van Pottelbergh G Dobbels F Daniels R Lauwerier E. Self-management among community-dwelling people with chronic conditions: adapting evidence-based group programs using intervention mapping. Patient Educ Couns. (2020) 103:589–96. doi: 10.1016/j.pec.2019.10.001 31704031

[90] Joodaki Z Mohammadzadeh S Salehi S. The relationship between job satisfaction and quality of life in nurses at Khorramabad educational hospitals, 2019. 2. J Nurs Educ. (2019) 8:25–32. [In Persian].

[91] Primanda Y Fatah DI. Knowledge and experience of community health volunteer (cadre) on type 2 diabetes mellitus Management in Yogyakarta. Open Access Maced J Med Sci. (2021) 9:240–4. doi: 10.3889/oamjms.2021.5863

[92] Goharinezhad S Maleki M Baradaran HR Ravaghi H. A qualitative study of the current situation of elderly care in Iran: what can we do for the future? Glob Health Action. (2016) 9:32156. doi: 10.3402/gha.v9.32156 27876456 PMC5120385

[93] Monjamed Z Ghorbani T Mostofian F Oveissipour R Nakhost Pandi S Mahmoudi M. A nationwide study of level of job satisfaction of nursing personnel in Iran. HAYAT. (2005) 10:39–48. [In Persian].

[94] Meng Y. Spiritual leadership at the workplace: perspectives and theories. Biomed Rep. (2016) 5:408–12. doi: 10.3892/br.2016.748 27699006 PMC5038601

[95] Montazer M Samim R Sedighyani A Mehdi R. Socio-cultural factors affecting the satisfaction of the elderly with retirement welfare (case study: Tehran municipality). Q Soc Stud Res Iran. (2019) 8:899–918. doi: 10.22059/jisr.2019.290055.943

[96] Nasrabadi AN Forooshani ZSD Rafiee F. Altruism the essense of the Iranian nurses’ job satisfaction: a qualitative study. Global J Health Sci. (2016) 8:13. doi: 10.5539/gjhs.v8n8p13 PMC5016361 27045394

[97] Liu X-L Willis K Fulbrook P Wu C-J Shi Y Johnson M. Factors influencing self-management priority setting and decision-making among Chinese patients with acute coronary syndrome and type 2 diabetes mellitus. Eur J Cardiovasc Nurs. (2019) 18:700–10. doi: 10.1177/1474515119863178, PMID: 31319694

[98] Mehri S Hosseini MA Fallahi-Khoshknab M Mohammadi Shahbelaghi F Akbari ZS. Clarification of ageism in the care system. Salmand Iran J Ageing. (2020) 15:14–27. doi: 10.32598/sija.2020.3.170

[99] Prasad C Davis KE Imrhan V Juma S Vijayagopal P. Advanced glycation end products and risks for chronic diseases: intervening through lifestyle modification. Am J Lifestyle Med. (2019) 13:384–404. doi: 10.1177/1559827617708991 31285723 PMC6600625

[100] Tsamlag L Wang H Shen Q Shi Y Zhang S Chang R. Applying the information–motivation–behavioral model to explore the influencing factors of self-management behavior among osteoporosis patients. BMC Public Health. (2020) 20:1–8. doi: 10.1186/s12889-020-8292-x 32028930 PMC7006415

[101] Harvey PW Petkov JN Misan G Fuller J Battersby MW Cayetano TN. Self-management support and training for patients with chronic and complex conditions improves health-related behaviour and health outcomes. Aust Health Rev. (2008) 32:330–8. doi: 10.1071/AH080330 18447824

[102] Delavar F Pashaeypoor S Negarandeh R. The effects of self-management education tailored to health literacy on medication adherence and blood pressure control among elderly people with primary hypertension: a randomized controlled trial. Patient Educ Couns. (2020) 103:336–42. doi: 10.1016/j.pec.2019.08.028 31451361

[103] Nguyen TNM Whitehead L Saunders R Dermody G. Systematic review of perception of barriers and facilitators to chronic disease self-management among older adults: implications for evidence-based practice. Worldviews Evid-Based Nurs. (2022) 19:191–200. doi: 10.1111/wvn.12563 35032152

[104] Byrne G Keogh B Daly L. Self-management support for older adults with chronic illness: implications for nursing practice. Br J Nurs. (2022) 31:86–94. doi: 10.12968/bjon.2022.31.2.86 35094539

[105] Zwerink M Brusse-Keizer M van der Valk PD Zielhuis GA Monninkhof EM van der Palen J. Self management for patients with chronic obstructive pulmonary disease. Cochrane Database Syst Rev. (2014) 3:2990. doi: 10.1002/14651858.CD002990.pub3 PMC7004246 24665053

[106] Wang X. Breaking the cycle of intergenerational trauma. Columbus, OH: The Ohio State University (2019).

[107] Hong QN Fàbregues S Bartlett G Boardman F Cargo M Dagenais P. The Mixed Methods Appraisal Tool (MMAT) version 2018 for information professionals and researchers. Education for information. (2018) 34:285–91.

[108] Dixon-Woods M Cavers D Agarwal S Annandale E Arthur A Harvey J. Conducting a critical interpretive synthesis of the literature on access to healthcare by vulnerable groups. BMC Med. Res. Methdol. (2006) 6:35. doi: 10.1186/1471-2288-6-35 PMC1559637 16872487

